# Supplementary material for: Harnessing the potential of spatial statistics for spatial omics data with pasta
Source: Nucleic Acids Res. 2025 Sep 10;53(17):gkaf870. doi: 10.1093/nar/gkaf870 (PMC12421381; doi:10.1093/nar/gkaf870)
Supplement: gkaf870_Supplemental_File [file gkaf870_supplemental_file.pdf]

Supplement to: "Harnessing the Potential of Spatial Statistics for Spatial Omics Data with *pasta*"

Martin Emons[1,†], Samuel Gunz[1,†], Helena L. Crowell[2], Izaskun Mallona[1], Malte Kuehl[3,4], Reinhard Furrer[5], and Mark D. Robinson[1,*]

[1]Department of Molecular Life Sciences and SIB Swiss Institute of Bioinformatics, University of Zurich, Zurich, Switzerland
[2]Centro Nacional de Análisis Genómico (CNAG), Barcelona, Spain
[3]Department of Clinical Medicine, Aarhus University, Aarhus, Denmark
[4]Department of Pathology, Aarhus University Hospital, Aarhus, Denmark
[5]Department of Mathematical Modeling and Machine Learning, University of Zurich, Zurich, Switzerland
[†]Equal contribution: both reserve the right to list themselves as first author; author order was determined by flipping a Swiss 5 franc coin.
[*]Correspondence to: `mark.robinson@mls.uzh.ch`

July 28, 2025

Supplementary data

Table S1: Non-exhaustive table of technologies and the data modality they can represent. For example, MERFISH can be represented as an irregular lattice using the outline of the cell segmentations. The cell centroids of the cell segmentations as well as the transcript locations can be represented as a point pattern.

**Technology**	**Main feature of interest**	**Lattice data**	**Point pattern**
Visium [12]	transcriptomics	spots (regular)	not applicable
Slide-seq V1& V2 [11, 13]	transcriptomics	beads (regular); segmented cells (irregular)	segmented cells
Visium HD [10]	transcriptomics	spots (regular); segmented cells (irregular)	segmented cells
Stereo-seq [3]	transcriptomics	spots (regular); segmented cells (irregular)	segmented cells
Xenium [8]	transcriptomics	segmented cells (irregular)	transcript locations; segmented cells
CosMx [3]	transcriptomics	segmented cells (irregular)	transcript locations; segmented cells
MERFISH [3]	transcriptomics	segmented cells (irregular)	transcript locations; segmented cells
IMC [5]	proteomics	pixels (regular); segmented cells (irregular)	segmented cells
MIBI-TOF [9]	proteomics	pixels (regular); segmented cells (irregular)	segmented cells
CODEX [2]	proteomics	segmented cells (irregular)	transcript locations; segmented cells
4i [6]	proteomics	segmented cells (irregular)	transcript locations; segmented cells

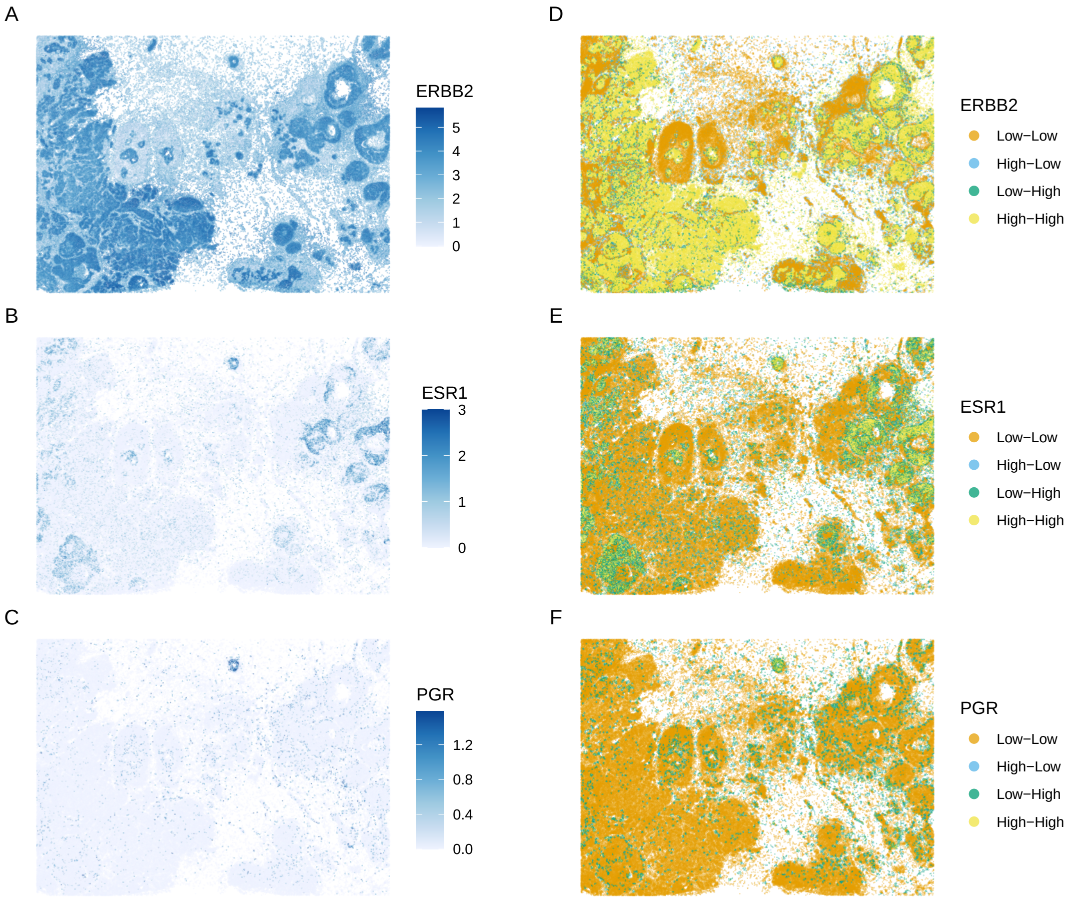

Figure S1: Spatial correlation (lattice data) analysis of three receptor genes (*ERBB2*, *ESR1* and *PGR*) in a Xenium human breast cancer data set [8]. **A-C**) The gene expression of three receptor genes *ERBB2*, *ESR1* and *PGR*. The neighbourhood of a location is defined as the 6 nearest neighbours. **D-F**) Moran's scatter plot to assess the type of spatial autocorrelation of the three genes.

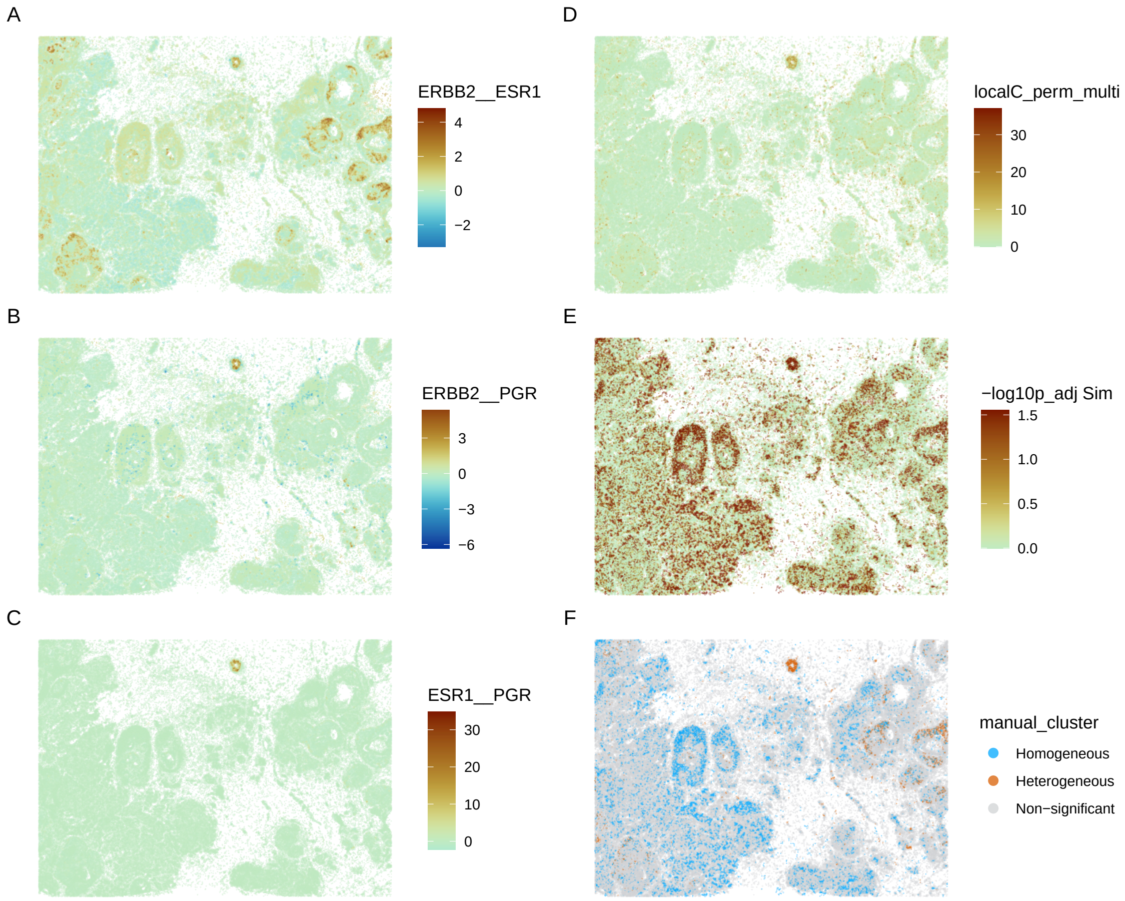

Figure S2: **A-C**) Bivariate Lee's L assessing spatial correlation for the pairwise combinations of the three genes. **D**) Multivariate Geary's c assessing spatial correlation for the three genes. Positive values indicate local heterogeneity. **E**) The corresponding adjusted p-values to the multivariate Geary's c on a log scale obtained from permutations. **F**) Multivariate Geary's c values were defined as homogeneous if $c < 1$ and heterogeneous if $c \geq 1$. Significance was determined by adjusted $p < 0.05$

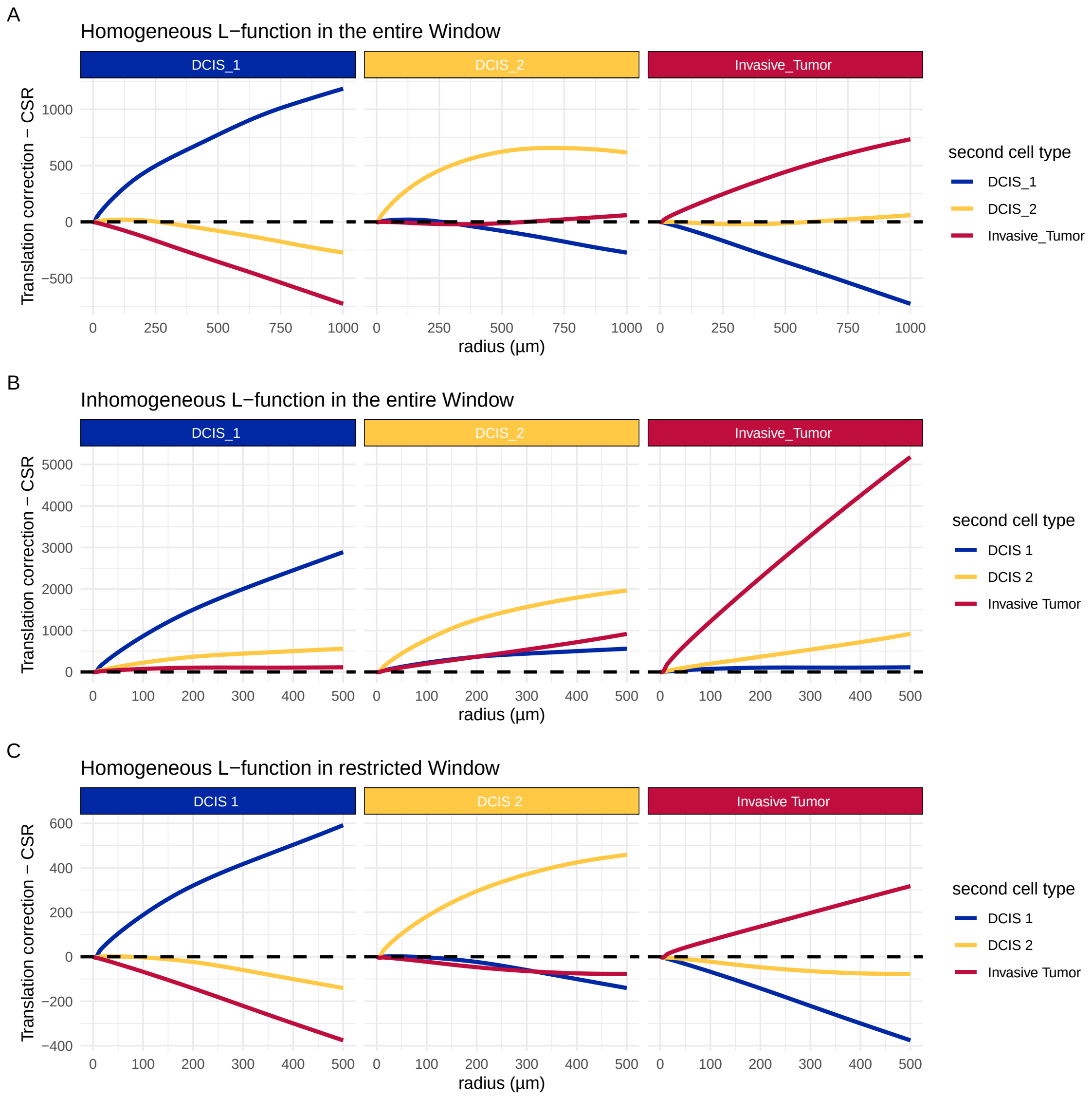

Figure S3: Three point pattern analyses of a Xenium human breast cancer data set [8]. Analysed are ductual carcinoma in situ 1 and 2, as well as invasive tumour cells. **A**) Homogeneous Besag's L function for the pairwise cell type combinations in the entire window, not correcting for any inhomogeneity in the distribution of cells in the tissue. **B**) Inhomogeneous Besag's L function for the pairwise cell type combinations in the entire window scaled by the average intensity of the unmarked point pattern per unit square. This corrects for the underlying inhomogeneous distribution of cells in the tissue. **C**) Homogeneous Besag's L function for the pairwise cell type combinations in a restricted window. The restricted window was determined with an intensity threshold on the unmarked point pattern.

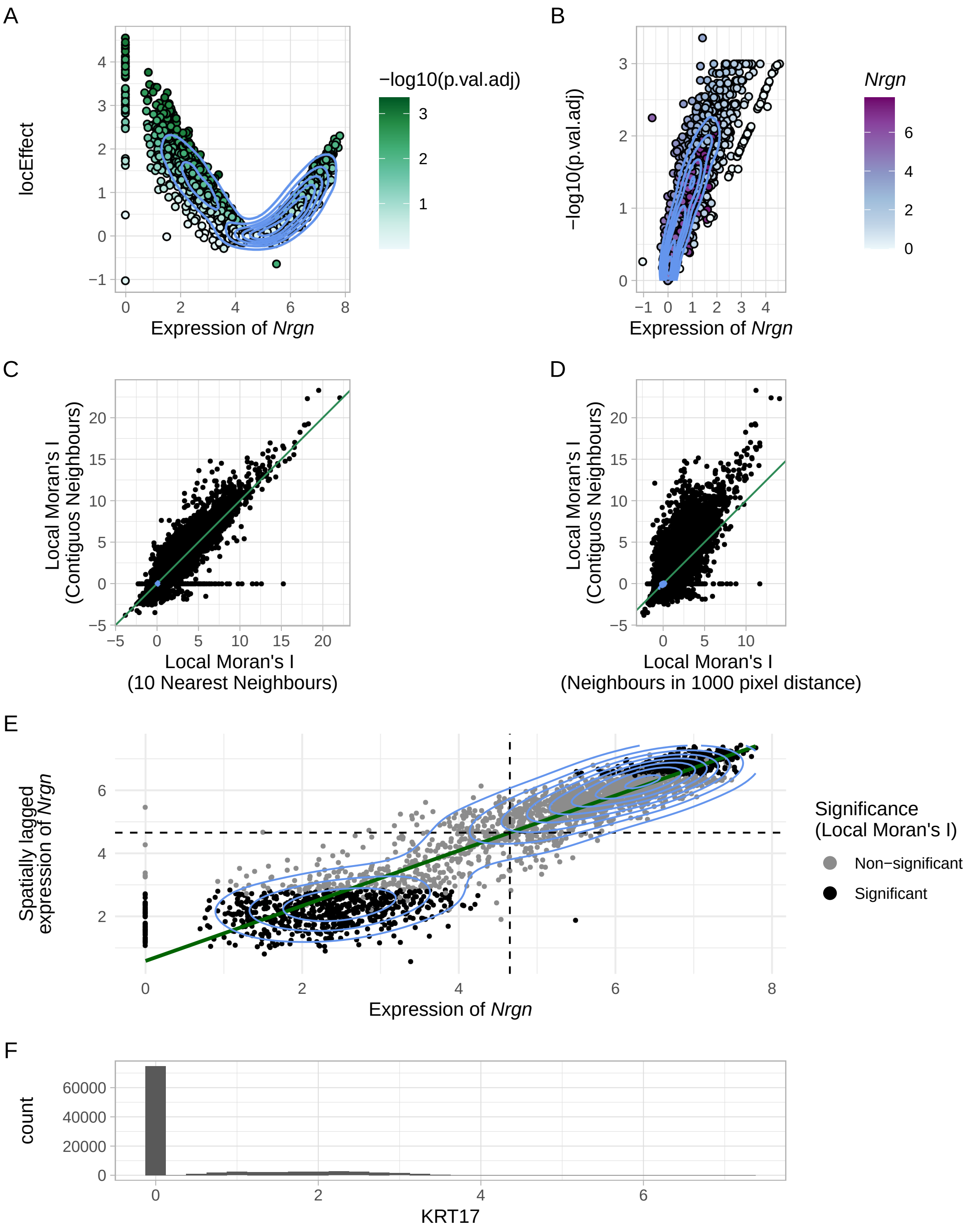

(Figure legend on next page.)

Figure S4: In **A-B**) each point represents a spot in the Visium mouse brain dataset [1]. Dependence between log-transformed counts of gene *Nrgn* and the corresponding local Moran's I values in A); the corresponding local Moran's I and adjusted p-values in B). The colours indicate the corresponding adjusted p-value in A) and log-transformed counts in B). **C-D**) show the relationship of local Moran's I values and the log-transformed counts of gene *KRT7* in the CosMx human non small cell lung cancer dataset [7]. Local Moran's I values calculated based on contiguous based neighbours vs. distance based neighbours in C); and contiguous based neighbours vs. neighbours in 1000 pixel distance in D). Red indicates cells with no contiguous neighbours. Green line indicates $x = y$. Blue lines indicate local densities. Note the high density close to the origin in C) and D) resulting from sparse expression of the gene *KRT7*, c.f., histogram in **F**). **E)** Moran's scatter plot displaying the expression of gene *Nrgn* in each spot vs. the average expression of its neighbours (as defined in the weight matrix). Significance values correspond to local Moran's I. The dotted lines correspond to the mean expression of *Nrgn* and are used to define clusters; c.f., Figure 5C)
.

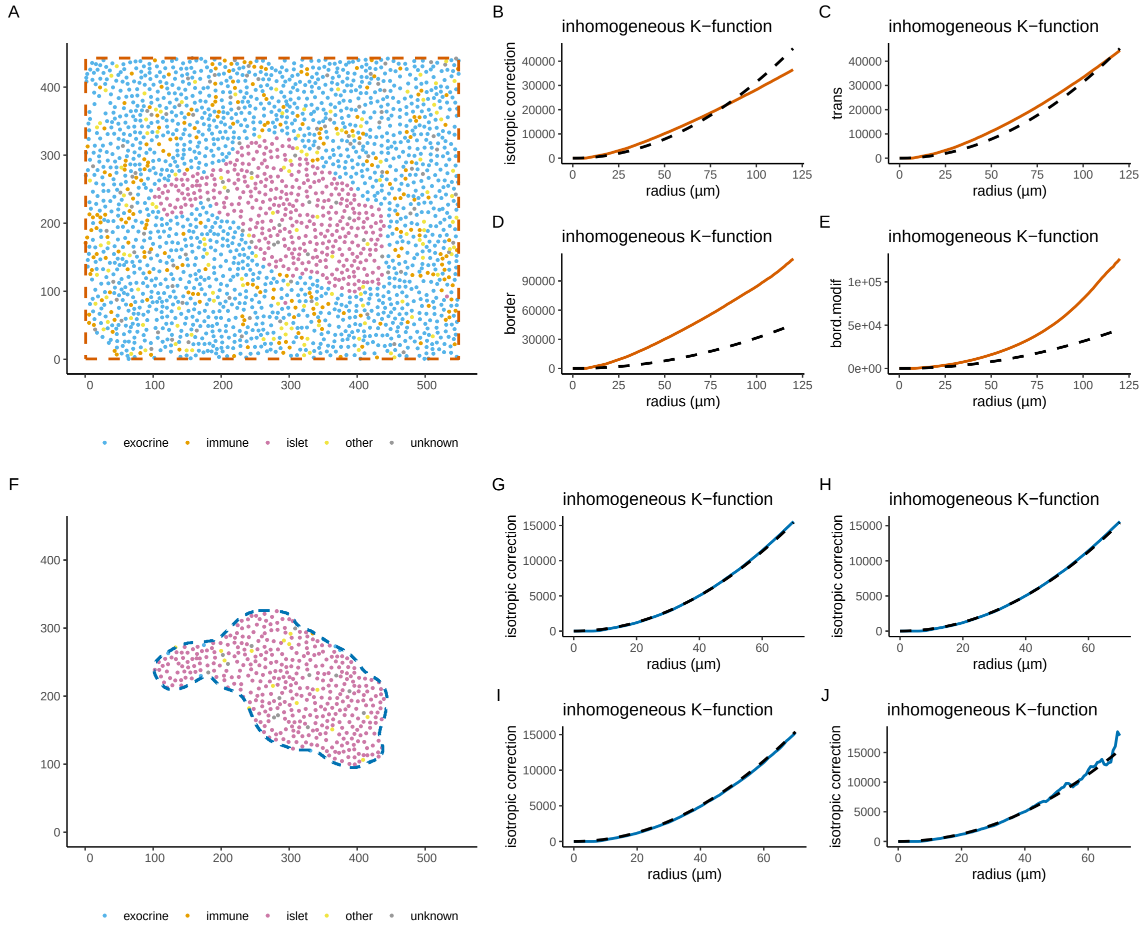

Figure S5: **A**) Single image of an IMC dataset showing islets in the human pancreas [4] The analysis window (dashed line) is set to correspond to the entire FOV. Global analysis using inhomogeneous K-function with **B**) isotropic, **C**) translational, **D**) border and **E**) modified border correction. **F**) Local analysis on subset of islet cells and window (dashed line) around tissue structure using inhomogeneous K-function with **G**) isotropic, **H**) translational, **I**) border and **J**) modified border correction.
